# Medically Documenting Disability in Myalgic Encephalomyelitis/Chronic Fatigue Syndrome (ME/CFS) Cases

**DOI:** 10.3389/fped.2019.00231

**Published:** 2019-07-02

**Authors:** Barbara B. Comerford, Richard Podell

**Affiliations:** ^1^Law Offices of Barbara Comerford, Paramus, NJ, United States; ^2^Robert Wood Johnson Medical School, Rutgers, The State University of New Jersey, New Brunswick, NJ, United States

**Keywords:** CFS, physician education, chronic fatigue sydrome, myalgic encephalomyelitis, ME

## Abstract

Patients with severe myalgic encephalomyelitis/Chronic fatigue syndrome (ME/CFS) experience debilitating physical and cognitive symptoms, which often result in the need to file disability claims. A significant number of ME/CFS patients are children or adolescents. ME/CFS patients often turn to physicians who are not trained to recognize and diagnose ME/CFS, and who might or might not understand that ME/CFS is a multi-system primarily physical illness. Such misperceptions can adversely affect the doctor-patient relationship, the clinical outcomes, as well as the results of disability claims. According to the National Academies of Science, Engineering and Medicine, “Between 836,000 and 2.5 million Americans suffer from myalgic encephalomyelitis/chronic fatigue syndrome… This disease is characterized by profound fatigue, cognitive dysfunction, sleep abnormalities, autonomic manifestations, pain, and other symptoms that are made worse by exertion of any sort. ME/CFS can severely impair patients' ability to conduct their normal lives.^1^” The prevalence of MECFS among children and adolescents has been estimated variously as between 0.11 and 4% (1). A large percentage of children and adolescents with ME/CFS suffer from orthostatic intolerance due to one or both of these syndromes: Neurally Mediated Hypotension (NMH) and Postural Orthostatic Tachycardia Syndrome (POTS). These elements of ME/CFS often respond well to proper treatment (2, 3).

## Medically Documenting a Social Security Disability Claim

Under the Social Security Regulations, a person is disabled if he is unable to engage in substantial gainful activity by reason of a medically determinable impairment which can be expected to last for a continuous period of not <12 months or result in death^2^. In April 2014, SSA issued updated guidelines for evaluating disability claims involving ME/CFS^3^. Social Security Ruling (SSR) 14-1p provides guidance on how to develop evidence and to establish that a person has a medically determinable impairment (MDI) of ME/CFS and explains how SSA evaluates those disability claims. Under 14-1p determination of an MDI includes a diagnosis of ME/CFS by a licensed physician using the CDC case definition for CFS, and to a lesser extent, the Canadian Consensus Criteria and the International Consensus Criteria supported by specific medical evidence consisting of signs, symptoms and lab findings. Physicians must therefore know what medical evidence is necessary for patients to qualify for Social Security Disability benefits. Under 14-1p licensed physicians must provide medical reports which include a “thorough medical history including onset, duration, diagnosis of CFS/ME, and the prognosis. Any co-morbid conditions should also be included. Treatment prescribed, the patient response and all clinical findings such as the results of physical and mental status exams, lab findings or any other clinically accepted form of objective testing should also be included. Medical signs observed by the physician including orthostatic intolerance, palpably swollen or tender axillary or cervical lymph nodes, persistent, reproducible muscle tenderness on exam, abnormal immune function, non-exudative pharyngitis” are a few examples^5^.

It is especially important to ask patients, including children and adolescents about orthostatic symptoms. At a minimum blood pressure and pulse should be documented lying down, sitting, immediately after standing, and then after remaining upright without moving for 2 to 5 min. Patients with orthostatic symptoms and/or substantial declines in blood pressure and/or tachycardia should be considered for specialty referral.

Physicians should also note symptoms or other effects of ME/CFS including: “persistent or relapsing fatigue resulting in reduction or impairment in the ability to carry out daily or work-related activities; post exertional malaise (worsening of symptoms after physical, cognitive or emotional effort); waking unrefreshed; disturbed sleep patterns; cognitive impairments (e.g., difficulty with information processing, short-term memory, reduced concentration and attention); persistent muscle pain, tenderness, stiffness, or weakness, multi-joint pain without swelling or redness; headaches of a new type, pattern or severity; frequent or re-occurring sore throats; cardiovascular abnormalities such as palpitations; gastrointestinal discomfort such as nausea, bloating, or abdominal pain; respiratory difficulties such as labored breathing or sudden breathlessness; urinary or bladder problems such as urinary frequency, nocturia, dysuria or pain in the bladder region or visual difficulties such as difficulty with focus, impaired depth perception or severe photosensitivity^6^.”

Physicians should also provide an opinion about the patient's ability to perform daily activities at home, at school or at work. For example, getting dressed in the morning, organizing the day's activities, concentrating at school or at work, the ability to sustain prolonged periods of walking, sitting, typing etc, and whether these activities often cause a prolonged flare up of symptoms and decline of function (Post Exertional Malaise/ PEM).

Most disability claims made on behalf of children and adolescents with ME/CFS are supplemental security income claims (SSI) claims. SSI claims are filed on behalf of disabled children whose parents' income fall below federal poverty levels^7^. And in many states children on SSI can qualify for Medicaid^8^.

Unlike adults, children with ME/CFS (and any other disabling conditions) must provide documentation of the existence of a medically determinable physical or mental impairment or impairments which result in *marked and severe functional limitations*; and that the impairment(s) lasted or can be expected to last for a continuous period of at least 12 months or be expected to result in death.

In determining whether a child experiences marked and severe functional limitations in the context of a child SSI case, SSA will consider proof^9^ regarding all the child's impairments, including their interactive and cumulative effects, and all the relevant information in (the child's) case record that helps determine functioning, including signs, symptoms, and laboratory findings, and the descriptions about functioning from the child's parents, teachers, and other people who know, and other relevant factors.

“The medical evidence may include formal testing that provides information about the child's development or functioning in terms of percentiles, percentages of delay, or age or grade equivalents. Standard scores (e.g., percentiles) can be converted to standard deviations. When such scores (are produced), (SSA) will consider them together with the information (SSA has) about (the child's) functioning to determine whether the child has a “marked” or “extreme” limitation in a domain^10^.

Children and adolescents can be diagnosed with ME/CFS if they suffer from the following symptoms:

- severe disabling fatigue that lasts for at least 3 months
- headaches
- sleep problems
- cognitive problems
- sore throat
- muscle aches and pains
- nausea, and dizziness
- Post-exertional malaise is a core symptom and the most useful when making a diagnosis^11^.

Children experience an increase in fatigue, malaise and symptoms after an increase in exertion. For many, this means they attend 1 or 2 days of school, before becoming too unwell to attend school at all. Some children are severely affected and post-exertional malaise presents as an increase in symptoms after, for example, taking a shower or walking down the stairs. Other symptoms that are almost universal in children and adults are cognitive dysfunction and disturbed/unrefreshing sleep^12^.”

Since there is no impairment listing for ME/CFS for either adults or children within the Code of Federal Regulations^13^ governing SS cases, both adult and children cases require documentation of severe functional limitations. For an impairment to equal the listings in an SSI child case through the domains of functioning, the ME/CFS must cause severe (marked) limitations that affect at least two of the six domains of functioning or an extreme limitation that affects at least one domain^14^.

SSA reviews a child/adolescent SSI case by examining whether there are marked and extreme functional deficits in one or more of six domains^15^. The Domains of Functioning evaluates a different area of functioning important in everyday life:

1. “Acquiring and using information^16^
2. Attending and completing tasks^17^
3. Interacting and relating with others^18^
4. Moving about and manipulating objects^19^
5. Caring for yourself^20^
6. Health and Physical well-being^21^.”

Under the SSA sixth domain of functioning, “Health and physical well-being,” to determine whether a child suffers from a “marked limitation,” it will consider whether a child is frequently ill because of her impairment(s) or has frequent exacerbations of his impairment(s) that result in significant, documented symptoms or signs. For purposes of this domain, “frequent” means that the child has episodes of illness or exacerbations that occur on an average of 3 times a year, or once every 4 months, each lasting 2 weeks or more. A Marked Limitation—is a limitation that severely interferes with a child's ability to engage in activities related to a domain of functioning. A marked limitation is more severe than a moderate limitation and is less severe than an extreme limitation^22^^,^^23^.

SSA may also find that the child has a “marked” limitation if he has episodes that occur more often than 3 times in a year or once every 4 months but do not last for 2 weeks, or occur less often than an average of 3 times a year or once every 4 months but last longer than 2 weeks, if the overall effect (based on the length of the episode(s) or its frequency) is equivalent in severity.

The functional limitations a child or adolescent who suffers from ME/CFS might experience “marked” limitation if the child is frequently ill because of the following symptoms: severe fatigue, lightheadedness, headaches, muscle aches and pains, sore throats, nausea, dizziness, sleep and cognitive deficits. If the child attends school but cannot sit the length of time other children can, and must lay down, that is a marked functional limitation most likely because of orthostatic intolerance. If the child is like that for weeks at a time, it is clearly a marked limitation. If the child is always like that, it may fall into the extreme category. And remember that under the functional child limitations, you need to document marked limitations in two domains or one extreme limitation in one domain. If the child cannot stay vertical for long because of orthostatic symptoms, that will impact the child's ability to engage in sports, or other school related activities. If the child has cognitive deficits, it is unlikely he will be able to participate in class as other children do. They may also require special testing with additional time due to slow processing speed. If they do participate in any of these activities, they may require extended rest, or miss days of school as a result of “crashes” also known as post exertional malaise.

Any diagnostics that can confirm these deficits are acceptable sources of medical evidence under SSR 14-1p.

To establish a disability, a child with ME/CFS (or any other disability) must also establish that she also suffers from a severe limitation. “Severe” in this context requires proof that the impairments very seriously interfere with a child's ability to engage in activities related to a domain of functioning. An extreme limitation is rather rare and is only given to the worst limitations.

A child or adolescent with ME/CFS can establish disability by demonstrating two marked limitations or one extreme limitation within these domains^24^.

Adult ME/CFS patients experience good days and bad days and post exertional malaise following exertion which impairs sustained, predictable function which significantly impacts the ability to perform any activities on a regular and sustained basis. Therefore, the physician should discuss good day/bad day and post exertional malaise presentation of symptoms. Cognitive deficits such as difficulties processing information, remembering, concentrating, and focusing should be addressed in the report. These same limitations exist in children.

Adult ME/CFS patients may also seek benefits under a disability insurance policy provided by an employer, or through purchase of a disability insurance policy from an insurance broker. Children and adolescents do not participate in the work force such that they are entitled to private long term disability insurance so the following discussion only pertains to adults.

## Medically Documenting a Long Term Disability Insurance Claim (LTD)

Some private employers offer short and long-term disability insurance coverage to eligible employees as part of an ERISA (Employee Retirement Income Security Act) welfare benefit plan. The LTD Plan provides a percentage of pre-disability income in the event the worker becomes disabled.

Individuals can also privately purchase disability income (DI) insurance directly through an insurance broker for a specific monthly benefit amount. The latter are not governed by ERISA, but rather state insurance laws.

Just as a physician must provide medical documentation for an ME/CFS SSD claim, she must also medically document the LTD claim. A patient must accurately report the ME/CFS symptoms/limitations to the physician to assist in devising a treatment plan and to clinically document the medical chart for both SSD and LTD claims.

## Medical Documentation for All Disability Claims

### ME/CFS Patients or Their Parents Should Keep a Daily Journal With Complaints and Limitations to Provide for the Physician at Each Office Visit

ME/CFS patients often complain of brain fog or other cognitive issues which may adversely impact reporting symptoms/limitations to the physician. The physician should request the patient keep a daily journal of complaints and limitations to ensure the medical chart documents the actual state of the patient's health, and the functional limitations which result. The symptom/limitation journal can therefore provide a more accurate picture of the patient's symptoms and limitations and, when given to the physician during a visit, becomes part of the chart for a physician to review for treatment purposes, and to answer questions posed by the disability insurance company or the Social Security Administration.

#### Good Day/Bad Day Constellation of Symptoms

ME/CFS patients often experience “good days” and “bad days” and a patient's chronicling activities on those days is important. For example, if taking a shower on a good day requires the ME/CFS patient to rest after for a period because he is exhausted, the journal can document that.

ME/CFS patients often define a “good day” as a day when she can perform one or two activities with rest intervals, hardly a good day to most people.

On bad days, the level of function can plummet to little more than eating, drinking and going to the bathroom. A contemporaneous description of such days is critical in this context, especially when LTD insurance companies frequently employ surveillance to undermine ME/CFS claims.

The U.S. General Accounting Office also conducts surveillance in Social Security Disability (SSD) claims. As a result, the patient journal entries may not simply put that surveillance in context, it can support disability.

For example, if an investigator records an ME/CFS claimant's outside the house performing a chore or two, the investigator might extrapolate from this brief period of normal activity that the person has the ability to sustain normal function throughout a full school or work day for 5 days each week. But if the claimant's journal entry on that day, records a post exertional flare up of physical pain, fatigue, or mental exhaustion, that documented evidence can undermine the insurer's attempt to pain the claimant's functional abilities in a false light. And often, good day activity is followed by physical and/or cognitive crashes which adversely impact the patient's function the next day. Therefore, the journal's description should continue for at least 24 h or more.

A common tactic of private LTD insurers is to schedule a medical exam by one of its medical vendors and then employ surveillance before and after to document the claimant's conduct. In ME/CFS cases, claimants often experience a worsening of symptoms following the insurance medical exam. A patient's journal entry can document what surveillance does not–including the worsening of symptoms during the hours or days following the exam.

The patient journal will also likely document the unpredictability of the symptoms and limitations from 1 day to the next, and often from 1 h to the next and the frequency of post exertional malaise when activities are performed.

### An ME/CFS Claimant Should not Participate in a Standard Functional Capacity Evaluation Scheduled by the LTD Insurer

Private LTD insurance companies often try to schedule standard functional capacity evaluations (FCE) (as opposed to a cardio pulmonary exercise test (CPET) performed in ME/CFS cases) to determine whether an ME/CFS claimant has the physical capacity to work. Use of the FCE for that purpose has been discredited for a variety of reasons ^25^.

On February 20, 2019, Richard Podell, M.D. conducted a search of the National Library of Medicine database searching keywords: Functional Capacity Evaluation, Chronic Fatigue Syndrome and located 11 citations and found there were zero studies published that claim to demonstrate any validity for the FCE as standardly used to predict or certify whether a person with chronic fatigue syndrome is well enough to be able to work. Dr. Podell has written extensively on the topic and has useful information on his website regarding documenting disability in ME/CFS cases^26^.

If an LTD carrier demands the ME/CFS claimant attend a standard FCE, a treating physician should prepare a letter explaining any deleterious effect that test will have on the patient's health. The most common effect of a prolonged standard FCE is post exertional malaise, and exacerbation of other physical and cognitive ME/CFS symptoms^27^.

It is a basic tenet of insurance law, that an insured is disabled when the activity in question would aggravate a serious condition affecting the insured's health. *Lasser v. Reliance Standard Life Ins. Co*., 344 F.3d 381 (3rd Cir. 2003). The treatise definition of disability holds that “[t]he insured is considered to be permanently and totally disabled when it is impossible to work without hazarding his or her health**…*,”****31 John Alan Appleman, Appleman on Insurance § 187.05[A]*, at 214 (2d ed.2007). *Lasser v. Reliance Standard Life Ins. Co., 146 F.Supp.2d 619, 628 (D.N.J. 2001) [Citing Herring v. Canada Life Assur. Co., 207 F.3d 1026 (8th Cir. 2000)]*.

### Whenever Possible, Objectively Document the Symptoms and Functional Limitations

The clinical longitudinal medical record is the first place the long-term disability insurer and SSA will look to determine the length of the illness, the signs and symptoms documented in the record, and what, if any, objective documentation of signs, symptoms and functional limitations exist. This is another reason why incorporating the patient journal entries into the medical record is critical in this context.

The primary concern of most treating physicians is to document and address patient signs and symptoms, not necessarily to record functional limitations. A hallmark of ME/CFS is debilitating fatigue and post exertional malaise (PEM)^28^. The LTD insurer will examine the medical record to determine whether the physician has recorded fatigue and PEM complaints during each visit, if medications were prescribed to address those complaints, the response, positive or negative the patient had to the medications, and any objective testing done to document it. The insurance reviewer will also often ask the treating physician to answer questions about the claimant's functional abilities.

Once again, the patient's journal record of complaints and functional limitations within the chart will give the physician the ability to reply to those questions.

In disability claims, medical documentation of physical exam findings during each visit, as well as, signs and symptoms, treatment plans, and objective test results often control the outcome of the claim. The patient chart charts the course of the claim^29^.

Cardio pulmonary exercise testing (CPET) is a diagnostic test ordered by some ME/CFS specialists to determine the extent of the functional limitations associated with fatigue and PEM. CPET testing is administered over 2 days when the patient pedals on a stationary bike while resistance is added incrementally. It monitors cardiovascular, respiration and recovery responses, workload, effort and metabolic response/oxygen consumption. Very often the MECFS patient performs significantly worse on day two, which objectively documents the existence of functionally limiting post-exertional malaise^30^.

While CPET is considered the “gold standard” to objectively document PEM in CFS/ME patients, it must be performed by a provider who understands ME/CFS to avoid misinterpreting the results.

ME/CFS patients often complain of many cognitive deficits including impaired information processing speed, decline in verbal fluency, memory and concentration issues^31^. The ME/CFS patient should be tested by a neuropsychologist familiar with ME/CFS to ensure the test results are accurately interpreted.

The more objective documentation of the ME/CFS patient complaints, the stronger the case. (with, for example the above tests and tilt table testing^32^, EEGs^33^, QEEGs^34^, SPECT scans^35^, PET scans^36^ MRIs^37^ etc.,)^38^ BEAM results indicated that the energy values of δ, θ, and α_1_ waves significantly increased in the observation group, compared with the control group (*P* < 0.05, *P* < 0.01, respectively), which suggests that the brain electrical activities in CFS patients were significantly reduced and stayed in an inhibitory state; 2) the increase of δ, θ, and α_1_ energy values in the right frontal and left occipital regions was more significant than other encephalic regions in CFS patients, indicating the region-specific encephalic distribution; 3) the correlation dimension in the observation group was significantly lower than the control group, suggesting decreased EEG complexity in CFS patients.

In ERISA LTD cases, the contents of the administrative record often determine whether the case is later won or lost not merely during the administrative appeal stage, but in litigation. Courts in ERISA LTD cases are often limited to determining whether the insurance claim reviewer abused its discretion in denying the claim. In such cases, the Court may not substitute its opinion for that of the insurance company unless an abuse of that discretion is found. As a result, if the LTD insurer ignores the substantial evidence of record, cherry picks only unfavorable evidence from the record to deny a claim, or otherwise fails to conduct a full and fair review of the claim, it may be found to have abused its discretion. For these reasons, the medical evidence of record in support of disability, and the ME/CFS claimant documentation of complaints and functional limitations incorporated into the medical chart are crucial. A similar review occurs in SSD cases.

### Provide a “Before and After” Record of Occupational and Everyday Functional Abilities

Disability insurers and SSA require all claimants to describe their work history, especially the occupational demands prior to disability onset. When a patient requests a physician respond to a disability inquiry or provide a report on their behalf, the physician should inquire about the patient's job demands to assess whether the patient's ME/CFS symptoms, limitations and restrictions reasonably prevent the patient from performing his own occupational demands or the demands of any occupation. If an ME/CFS claimant was physically active and engaged prior to disability, but has abandoned all or many of those activities, that should also be documented in the medical record.

## Conclusion

The ME/CFS claimant must document the total adverse effect the constellation of symptoms has on his/her functional abilities and should provide that documentation in journal form to his treating physician during each visit. No claim can succeed without medical support and documentation of symptoms and functional limitations (physical and cognitive) by informed ME/CFS medical providers. The ME/CFS claimant medical record of functional limitations, and objective documentation of those limitations provided by the treating physician is crucial to support the ME/CFS disability claim.

If keeping a daily journal is not practical, we recommend that the patient or parent at each doctor visit submit 3 or 4 recent real life examples of episodes when the patient did “too much,” how the symptoms then flared and functional abilities declined, and how many hours or days were needed before symptoms and functional abilities regained their pre-exertional baselines.

## Author's Note

BC, Esq has been practicing disability insurance law, and Social Security Disability law since 1985 (www.tristatedisabilitylaw.com). She has lectured extensively and presented papers on these topics to lawyers, judges and disability organizations around the nation. Recently, she and her senior associate, Sara Kaplan-Khodorovsky, successfully represented a Washington Post reporter disabled with ME/CFS in an ERISA LTD lawsuit against Prudential Insurance Company. See Vastag v. Prudential Ins. Co. of Am., 2018 WL 2455921 (D.N.J. May 31, 2018). RP, M.D., MPH serves as clinical professor in the Department of Family Medicine at Rutgers-Robert Wood Johnson Medical School and as a Visiting Investigator at Rockefeller University.

## Author Contributions

All authors listed have made a substantial, direct and intellectual contribution to the work, and approved it for publication.

### Conflict of Interest Statement

The authors declare that the research was conducted in the absence of any commercial or financial relationships that could be construed as a potential conflict of interest. The handling Editor declared a shared affiliation at the time of review, though no other collaboration, with one of the authors RP.

## Footnotes

^2^See SSR 14-1p.

^3^SSR 14-1p “CFS is a systemic disorder consisting of a complex of symptoms that may vary in frequency, duration, and severity…”

^5^Id.

^6^Id.

^7^https://www.ssa.gov/ssi/text-child-ussi.htm. WHO IS A “CHILD” FOR SSI? A person who is neither married (as determined by Social Security) nor head of a household and: under age 18; or under age 22 and is a student regularly attending school (as determined by Social Security). To be eligible for SSI benefits, a child must be either blind or disabled. A child may be eligible for SSI disability benefits beginning as early as the date of birth; there is no minimum age requirement. A child may be eligible for SSI disability benefits until attainment of age 18 (see definition of disability for children). When the child attains age 18, we evaluate impairments based on the definition of disability for adults (see definition of disability for adults). The child has a medically determinable physical or mental impairment or impairments ***which result in marked and severe functional limitations; and*** The impairment(s) has lasted or can be expected to last for a continuous period of at least 12 months or be expected to result in death; or

^8^If a child is under age 18, not married, and lives at home with parent(s) who do not receive SSI benefits, we may consider a portion of the parents' income and resources as if they were available to the child. We may also count a portion of a stepparent's income and resources if the child lives with both a parent and a stepparent (or an adoptive parent and a stepparent). We also do this when a child is temporarily away at school, returns home during weekends, holidays or during the summer and remains subject to parental control. We call this process “deeming”. We make deductions from deemed income for parents and for other children living in the home. After we subtract these deductions, we use the remaining amount to decide if the child meets the SSI income and resource requirements for a monthly benefit. Deeming from the parent stops when a child attains age 18, marries, or no longer lives with a parent. Deeming does not apply, and we may pay up to $30 plus the applicable State supplement when: a disabled child receives a reduced SSI benefit while in a medical treatment facility; and the child is eligible for Medicaid under a State home care plan; and deeming would otherwise cause ineligibility for SSI benefits. Also, we do not consider the income of a parent for deeming purposes if the parent receives a Public Income Maintenance payment (PIM) such as Temporary Assistance for Needy Families (TANF) and his or her other income was used to compute the PIM payment. If either child or parent is temporarily absent from the household (less than 60 days), the rules about deemed income still apply. In most States, a child who gets SSI benefits can get Medicaid to help pay medical bills. In some cases, a child may be eligible for Medicaid while in an institution, but not be eligible when living at home either because of the parents' income and resources or because of other income. At the State's option, children under age 18 who need institutional–level care and live at home may keep Medicaid eligibility while getting home care, if that care is less costly to the government. Even if the child is not eligible for SSI benefits, the child still may be eligible for Medicaid under other State rules. Always check on Medicaid eligibility with the State. For more information about Medicaid, you can look on the internet on the Centers for Medicare & Medicaid Services website at http://www.medicaid.gov/index.html or call toll–free, 1–800–633–4227. In addition, other State services may also be available.

^9^https://secure.ssa.gov/poms.nsf/lnx/0425225020

^10^https://secure.ssa.gov/poms.nsf/lnx/0425225020 “We will find that you have a “marked” limitation in a domain when your impairment(s) interferes seriously with your ability to independently initiate, sustain, or complete activities. Your day-to-day functioning may be seriously limited when your impairment(s) limits only one activity or when the interactive and cumulative effects of your impairment(s) limit several activities. “Marked” limitation also means a limitation that is “more than moderate” but “less than extreme.” It is the equivalent of the functioning we would expect to find on standardized testing with scores that are at least two, but less than three, standard deviations below the mean. If you have not attained age 3, we will generally find that you have a “marked” limitation if you are functioning at a level that is more than one-half but not more than two-thirds of your chronological age when there are no standard scores from standardized tests in your case record. If you are a child of any age (birth to the attainment of age 18), we will find that you have a “marked” limitation when you have a valid score that is two standard deviations or more below the mean, but less than three standard deviations, on a comprehensive standardized test designed to measure ability or functioning in that domain, and your day-to-day functioning in domain-related activities is consistent with that score. (See DI 25225.020D). ***For the sixth domain of functioning, “Health and physical well-being,” we may also*** ***consider you to have a “marked” limitation if you are frequently ill because of your*** ***impairment(s) or have frequent exacerbations of your impairment(s) that result in*** ***significant, documented symptoms or signs. For purposes of this domain, “frequent”*** ***means that you have episodes of illness or exacerbations that occur on an average of 3*** ***times a year, or once every 4 months, each lasting 2 weeks or more. We may also find that*** ***you have a “marked” limitation if you have episodes that occur more often than 3 times in*** ***a year or once every 4 months but do not last for 2 weeks, or occur less often than an*** ***average of 3 times a year or once every 4 months but last longer than 2 weeks, if the overall*** ***effect (based on the length of the episode(s) or its frequency) is equivalent in*** ***severity*.** C. Policy – Extreme Limitation ***We will find that you have an “extreme” limitation in a domain when your*** ***impairment(s) interferes very seriously with your ability to independently initiate, sustain*,** ***or complete activities. Your day-to-day functioning may be very seriously limited when your*** ***impairment(s) limits only one activity or when the interactive and cumulative effects of*** ***your impairment(s) limit several activities. “Extreme” limitation also means a limitation*** ***that is “more than marked.” “Extreme” limitation is the rating we give to the worst*** ***limitations. However, “extreme limitation” does not necessarily mean a total lack or loss of*** ***ability to function. It is the equivalent of the functioning we would expect to find on*** ***standardized testing with scores that are at least three standard deviations below the*** ***mean*.** If you have not attained age 3, we will generally find that you have an “extreme” limitation if you are functioning at a level that is one-half of your chronological age or less when there are no standard scores from standardized tests in your case record. If you are a child of any age (birth to the attainment of age 18), we will find that you have an “extreme” limitation when you have a valid score that is three standard deviations or more below the mean on a comprehensive standardized test designed to measure ability or functioning in that domain, and your day-to-day functioning in domain-related activities is consistent with that score. (See DI 25225.020D). ***For the sixth domain of functioning, “Health and physical well-being,” we may also*** ***consider you to have an “extreme” limitation if you are frequently ill because of your*** ***impairment(s) or have frequent exacerbations of your impairment(s) that result in*** ***significant, documented symptoms or signs substantially in excess of the requirements for*** ***showing a “marked” limitation in DI 25225.020B.4. However, if you have episodes of*** ***illness or exacerbations of your impairment(s) that we would rate as “extreme” under this*** ***definition, your impairment(s) should meet or medically equal the requirements of a listing*** ***in most cases. See DI** 25220.010*.**

^11^https://www.ncbi.nlm.nih.gov/pmc/articles/PMC5919160/

^12^https://www.ncbi.nlm.nih.gov/pmc/articles/PMC5919160/

^13^CFR Part 404, Subpart P Appendix 1.

^14^https://www.disabilitycarecenter.org/medical-qualifications/domains-of-functioning/

^15^https://www.disabilitycarecenter.org/medical-qualifications/domains-of-functioning/

^16^Acquiring and Using Information This domain considers how well a child can learn and acquire information as well as their ability to utilize the information. From birth, children start learning and acquiring information through exploring the world and receiving formal education by attending school. As they grow up, children should adapt and acquire skills in communication, arithmetic, reading, writing, and reasoning through their experiences. These skills should progress with complexity as they age and eventually can be utilized in a workplace or community environment. A child may have a marked or extreme limitation in this domain if he or she: He or she cannot show understanding of works related to size, space, or time He or she is unable to rhyme words He or she has difficulty remembering important concepts learned the day beforehand He or she has difficulty solving math or arithmetic problems He or she talks in simple, short sentence and has trouble explaining what they mean

^17^https://www.disabilitycarecenter.org/medical-qualifications/domains-of-functioning/ Attending and Completing Tasks This domain evaluates a child's ability to focus and maintain attention as well as their ability to begin, continue, and complete activities at a normal pace for their age. Typically a child should be able to regulate alertness, filter out distractions, and maintain focus on a task or activity. While attending school, these abilities are critical for a child to effectively follow instructions, keep organized, and complete assignments. A child may have a marked or extreme limitation if he or she: Is easily startled, distracted or overreacts to touch, sounds, or movements Is slow to focus on, or unable to finish activities of interest Frequently becomes sidetracked from activities or repeatedly interrupts other Becomes frustrated easily and gives up on tasks Requires additional supervision to maintain engagement in an activity

^18^https://www.disabilitycarecenter.org/medical-qualifications/domains-of-functioning/ Interacting and Relating to Others This domain focuses on a child's ability to connect and cooperate with others, abide by rules or restrictions, respond to authority or criticism, respect the possessions of others, and develop a sense of community. A child should develop close personal relationships with family and friends, and work cooperatively with other children in school or the community. A child should also understand and respect social rules in various environments such as what is behavior is acceptable at home compared to being in public at a grocery store. A child may have a marked or extreme limitation if he or she: Does not reach out to be picked up by his or her guardians Has no close friends of the same age Avoids contact with others including people they know Has problems with adequate fluency when speaking Has difficulty engaging in activities with rules (such as board games or sports) Has difficulty with communication; He or she struggles with expressing emotions, continuing a conversation, or asking for help

^19^https://www.disabilitycarecenter.org/medical-qualifications/domains-of-functioning/ Moving About and Manipulating Objects This domain involves how well a child can move his or her body from one location to another and how he or she moves and manipulates objects. These movements are known as “gross” and “fine” motor skills and are described below. Gross and Fine Motor Skills Gross motor skills involve movement and coordination of the limbs (arms and legs) and other large body parts while fine motor skills refer to the movement and coordination of smaller body parts such as the hands, fingers, wrists, feet, ankles and toes. Gross motor skills include movements such as running, kneeling, bending, and crawling. Fine motor skills include movements such as grasping, gripping, and writing. The physical capabilities of a child depend on his or her age. A 6 year old is not expected to have the same complex motor skills as a 16 year old. As children age, they should develop more complex motor skills appropriate for their age. Below are some examples of limitations for this domain. A child may have a marked or extreme limitation if he or she: Experiences sensory loss, muscle weakness, or joint stiffness Has difficulty keeping balance, climbing stairs, or maintaining organized locomotion Has trouble with coordinating gross motor movement Has trouble with fine motor movement Has difficulty with complex finger or hand movements Has poor hand-eye coordination while using scissors or a pencil

^20^https://www.disabilitycarecenter.org/medical-qualifications/domains-of-functioning/ Caring for Self This domain evaluates how well a child is able to take care of him or herself. This includes a child's ability to meet his or her emotional and physical wants and needs as well as how the child deals with changes in his or her environment. As children grow, they should learn to understand how to regulate themselves independently and take care of their own personal needs, possessions, health, and safety (appropriate for their age). A child may have a marked or extreme limitation if he or she: Repeatedly places inedible objects in his or her month Consistently engages in self-soothing activities exhibiting developmental regression Is unable to dress or bathe properly for his or her age Often engages in self-harming behavior or disregards safety rules Does not seek out activities of interest Has disturbed sleeping and eating patterns

^21^Id.

^22^https://www.disabilitycarecenter.org/medical-qualifications/domains-of-functioning/ Health and Physical Well-Being This domain considers the cumulative physical effects of the child's impairment(s) and its associated medications or treatments that are not considered in the “Caring for Self” domain. Physical and mental disabilities can have physical effects that vary in severity and can inhibit a child's ability to perform activities effectively or independently. The medication a child takes for his or her disability may potentially create physical side effects that interfere with daily activities. A child may have a marked or extreme limitation if he or she: Needs intensive medical care to maintain health and well-being Physical limitations manifested from treatments, medication, or surgeries Has generalized symptoms caused by his or her condition such as dizziness, lethargy, weakness, agitation, or psychomotor retardation.

^23^https://www.disabilitycarecenter.org/medical-qualifications/domains-of-functioning/

^24^https://www.ssa.gov/disability/professionals/bluebook/evidentiary.htm

^25^https://www.aafp.org/afp/2007/0715/p247.html and https://www.prohealth.com/library/functional-capacity-evaluation-fce-and-your-disability-insurance-benefits-33900

^26^http://www.fmnetnews.com/coping-resources/disability-issues, https://www.drpodell.org/

^27^Id.

^28^https://www.ncbi.nlm.nih.gov/pmc/articles/PMC5983853/

^29^https://law.justia.com/cases/federal/district-courts/new-jersey/njdce/2:2015cv06197/323449/44/

^30^https://www.ncbi.nlm.nih.gov/pmc/articles/PMC4482824/ and http://neuroimmune.cornell.edu/research/physiology/; https://www.ncbi.nlm.nih.gov/pmc/articles/PMC4734796/

^31^https://www.ncbi.nlm.nih.gov/pmc/articles/PMC3655280/ and http://www.ncf-net.org/library/sp.htm

^32^http://iacfsme.org/ME-CFS-Primer-Education/Bulletins/2010/Results-of-Head-Upright-Tilt-Table-Test-Full-Artic.aspx

^33^https://www.ncbi.nlm.nih.gov/pmc/articles/PMC4734796/

^34^https://books.google.com/books?id=g_xcAgAAQBAJ&pg=PA54&lpg=PA54&dq=CFS+patients+tested+with+QEEGs&source=bl&ots=bBcUSL7DJy&sig=SSGbTXul6il-Zmhi1FFZAkvCQYY&hl=en&sa=X&ved=2ahUKEwir_Z7Z-tPdAhWDNd8KHaDkD3gQ6AEwBHoECAYQAQ#v=onepage&q=CFS%20patients%20tested%20with%20QEEGs&f=false

^35^http://www.spl.harvard.edu/publications/item/view/1351

^36^https://www.ncbi.nlm.nih.gov/pmc/articles/PMC5111735/

^37^https://www.ncbi.nlm.nih.gov/pmc/articles/PMC5633338/

^38^http://www.ncf-net.org/library/sp.htm Patients with chronic fatigue syndrome and depression had similar numbers of defects per patient (6.53 and 6.43, respectively). In all groups, defects were located predominantly in the frontal and temporal lobes. The midcerebral uptake index was found to be significantly lower (*p* <0.002) in the patients with chronic fatigue syndrome (0.667) and patients with AIDS dementia complex (0.650) than in patients with major depression (0.731) or healthy control subjects (0.716). Also, a significant negative correlation was found between the number of defects and midcerebral uptake index in patients with chronic fatigue syndrome and AIDS dementia complex, but not in depressed patients or control subjects.
